# Clocks for all seasons: unwinding the roles and mechanisms of circadian and interval timers in the hypothalamus and pituitary

**DOI:** 10.1530/JOE-14-0141

**Published:** 2014-08

**Authors:** Shona Wood, Andrew Loudon

**Affiliations:** Faculty of Life Sciences, University of Manchester, Manchester, M13 9PT, UK

**Keywords:** pars tuberalis, melatonin, Eya3, thyroid hormone, thyrotrophin, photoperiod, circadian

## Abstract

Adaptation to the environment is essential for survival, in all wild animal species seasonal variation in temperature and food availability needs to be anticipated. This has led to the evolution of deep-rooted physiological cycles, driven by internal clocks, which can track seasonal time with remarkable precision. Evidence has now accumulated that a seasonal change in thyroid hormone (TH) availability within the brain is a crucial element. This is mediated by local control of TH-metabolising enzymes within specialised ependymal cells lining the third ventricle of the hypothalamus. Within these cells, deiodinase type 2 enzyme is activated in response to summer day lengths, converting metabolically inactive thyroxine (T_4_) to tri-iodothyronine (T_3_). The availability of TH in the hypothalamus appears to be an important factor in driving the physiological changes that occur with season. Remarkably, in both birds and mammals, the pars tuberalis (PT) of the pituitary gland plays an essential role. A specialised endocrine thyrotroph cell (*TSH*-expressing) is regulated by the changing day-length signal, leading to activation of TSH by long days. This acts on adjacent TSH-receptors expressed in the hypothalamic ependymal cells, causing local regulation of deiodinase enzymes and conversion of TH to the metabolically active T_3_. In mammals, the PT is regulated by the nocturnal melatonin signal. Summer-like melatonin signals activate a PT-expressed clock-regulated transcription regulator (*EYA3*), which in turn drives the expression of the TSHβ sub-unit, leading to a sustained increase in TSH expression. In this manner, a local pituitary timer, driven by melatonin, initiates a cascade of molecular events, led by *EYA3*, which translates to seasonal changes of neuroendocrine activity in the hypothalamus. There are remarkable parallels between this PT circuit and the photoperiodic timing system used in plants, and while plants use different molecular signals (constans vs *EYA3*) it appears that widely divergent organisms probably obey a common set of design principles.

## Introduction

In virtually all habitats, even the tropics, there are marked seasonal changes in temperature, precipitation and food availability. This provides a powerful selection pressure, which has led to the evolution of long-term timing mechanisms allowing organisms to predict key environmental changes. Mammals exhibit a remarkably wide spectrum of seasonal physiological adaptations, which includes annual cycles of growth, metabolism, thermogenesis, fattening and weight loss, hibernation, migration, moulting and pelage growth, and sexual behaviour, all of which are synchronised by internal timing mechanisms and provide an adaptive seasonal programme. This adaptive seasonal programme can be remarkably precise, for example, the median onset of birth dates for the arctic caribou varies by less than 4 days/annum (Post & Forchhammer 2008). Many of our domesticated species retain their seasonal ancestry for many processes, including reproduction, growth and moult cycles, and this remains a dominant feature of most animal production systems around the world.

Seasonal changes in food availability and temperature might be predicted to serve as cues, timing activation of reproductive and other neuroendocrine circuits in birds and mammals. However, there is now overwhelming evidence that seasonal changes in day length (photoperiod) provide the primary environmental cue for a diverse range of organisms. In contrast to all other environmental cues, photoperiod offers a highly predictive signal that can be reliably used by both plants and animals to activate reproductive and growth processes at the most appropriate time of year. In small mammals with short gestation periods, breeding typically occurs in response to increasing day lengths in the springtime (‘long-day (LD)’ breeders). But with a progressive rise in body size, and longer gestation periods, larger mammals such as sheep and deer need to mate in the autumn (‘short-day’ breeders).

## The photoperiodic response

**Figure 1:**
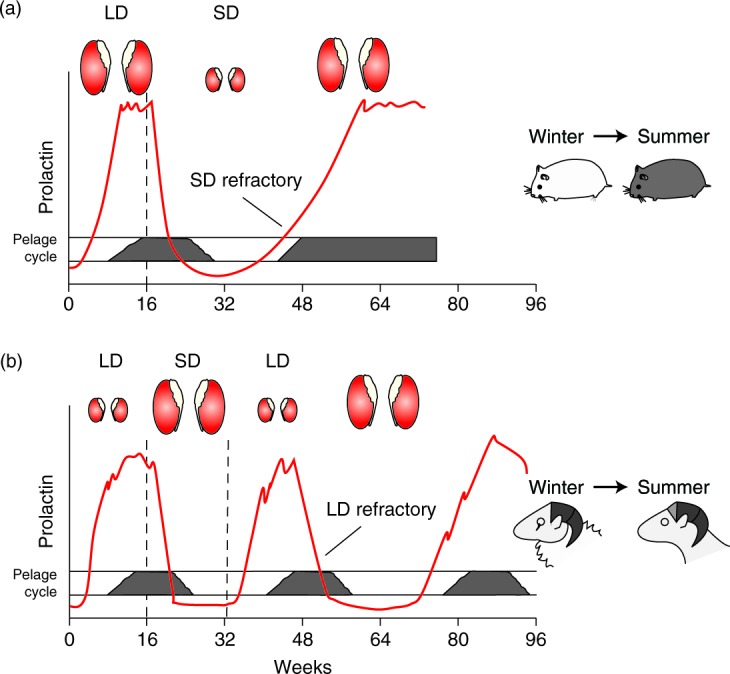
The seasonal prolactin rhythm and the associated pelage/moult and testis cycle in (a) male Siberian hamster and (b) male sheep exposed to long days (LD, 16 h light:8 h darkness) and short days (SD, 8 h light:16 h darkness). LD activates and SD inhibits prolactin release, driving pelage/moult responses. In the sheep, SD exposure activates the reproductive axis and LD leads to regression. Conversely in the hamster, LD activates the reproductive axis, and SD exposure leads to regression. Prolonged SD results in the development of photo-refractoriness with prolactin secretion resuming from 16 to 38 weeks (SD refractory state). This reversion to an LD-like state leads the coat to change from the white, winter pelage to the agouti, summer pelage and activation of the testis. In sheep, exposure to prolonged LD results in an initial refractory response from 12 to 36 weeks (LD refractory state). This merges into the expression of the circannual cycle; horn growth is suppressed during the winter leaving a permanent record of the cycles as rings in the horns. Adapted from Lincoln *et al*. (2003), first printed in *Journal of Endocrinology*.

The photoperiodic response of seasonal animals is known to involve a mechanism for registering changes in day length, and translating them into a neuroendocrine response. This ‘photo-neuroendocrine system’ was first defined as such by Berte and Ernst Scharrer in 1964 (Korf *et al*. 1998) and is a recognised universal feature of vertebrate and invertebrate responses to environmental change. The process of photo-induction is genetically programmed and driven by a conserved molecular mechanism in all tetrapods. A typical robust read-out of the seasonal response is seen in the expression of a seasonal pituitary prolactin rhythm, which is activated by long photoperiods, driving moult cycles in birds and mammals (Fig. 1). In small short-lived mammals such as Siberian hamsters, LD-activated prolactin secretion is suppressed by exposure to short days (SD), leading to marked changes in pelage and development of a white winter coat, but following prolonged exposure to SD, prolactin concentrations rise (i.e. the photo-neuroendocrine system becomes ‘refractory’) restoring the dark agouti summer coat in hamsters. Such responses are a universal feature of photoperiodic species, in which the initial response to photoperiod reverts over many weeks or months leading to a reversal of phenotype (Fig. 1). This refractory mechanism is common to virtually all seasonally breeding mammals that are sensitive to photoperiod change, including marsupial lineages (Brinklow & Loudon 1993).

In many small short-lived species (e.g. hamsters), refractoriness to SD signals is a widespread feature, but once established, animals remain ‘locked’ into a LD phenotype irrespective of how long they are exposed to SD signals. It is important to note that the reproductive rhythm is species-specific, and not phased to that of prolactin (i.e. in hamsters, reproductive activation and prolactin secretion are co-incident as both are springtime related). In long-lived species, long-term rhythms are established following prolonged exposure to fixed photoperiods, translating into cycles of neuroendocrine regulation of approximately 1 year in duration. These have been termed ‘circannual’ cycles and are a recognised feature of the biology of all long-lived vertebrate species which breed over several different seasons (Gwinner 1981, Woodfill *et al*. 1994). This is exemplified in the seasonally breeding sheep by the generation of long-term rhythms of prolactin, which drive the moult cycle (Fig. 1). In many seasonal species, including sheep and birds (Gwinner & Dittami 1990), the generation of sustained long-term rhythms requires exposure to LD conditions, and generally circannual cycles do not emerge unless animals are housed in artificial summer day lengths. Thus, a photoperiodic read-out is clearly a requirement for generation of circannual rhythm in such animals (Lincoln *et al*. 2006, Dardente 2012). We return to this issue below when we consider some of the molecular mechanisms known to drive LD responses, and their regulation by the circadian clock. Circannual rhythms are also dominant characteristic of the reproductive biology of many tropical species. For instance, even when species such as the tropical axis deer are transferred to temperate environments, they retain persistent long-term rhythmicity in antler and testicular cycles, which are not synchronised with others in same population, or to external photoperiod (Loudon & Curlewis 1988).

Seasonal variation in human hair growth and shedding (moulting) has been described; however these have not been related to seasonal variation in prolactin (Randall & Ebling 1991). Seasonal variation in plasma testosterone in men has also been observed and suggested to be related to hair growth cycles (Randall & Ebling 1991). Further evidence of seasonal cycles in humans has been noted in the seasonal variation in births; however, neuroendocrinologists do not agree that that reproduction is photo-responsive in humans (Bronson 1995).

The processes responsible for the generation of the circannual rhythm remains a subject of considerable interest to the biological timing field, but to date, rather little is yet known of the mechanisms involved, and the topic has recently been reviewed (Hazlerigg & Lincoln 2011). Finally, in commonly used laboratory species such as mice, seasonal physiological responses are absent, and the reproductive system remains active irrespective of the photoperiod. But fascinatingly, aspects of the upstream signalling system driving seasonal neuroendocrine function remain intact, and this has been exploited in studies which have explored the genetic basis of seasonal timing.

## How circadian clocks might time seasonal reproduction: Bünning's hypothesis

Photoperiodism implies that an organism must be able to discriminate either the length of light or nocturnal phase, or both. In 1936, Erwin Bünning, using both plant and insect models, developed the ground-breaking concept that photoperiodic species might use the endogenous timing system of the daily circadian clockwork. He proposed a light-requiring phase (photophil) of approximately 12 h, and a dark-requiring phase (scotophil) of approximately 12 h, which both combine to a 24 h period (Bünning 1936). If light is only experienced in the photophil, then a SD response is triggered. A variant of this hypothesis was proposed by Colin Pittendrigh and colleagues (the internal co-incident timing model), in which light's only role is to entrain a multi-oscillator circadian system, with the phase of the dawn and dusk oscillators being set by the length of the photoperiod. Each of the oscillators will behave in a different manner, depending on the light–darkness cycle and assume different phase-relationships with the entraining cycle. Changes in the ‘internal’ co-incidence of these oscillators would then determine the photoperiodic response (Pittendrigh & Minis 1964).

The Bünning's hypothesis and the later model of Pittendrigh are now widely accepted as the basis for photoperiodic time measurement in birds and mammals. The key experimental proof was provided by results from a series of studies reported by Nanda & Hamner (1958) on the SD flowering response of soybean plants, in which 8 h light cycles were combined with nocturnal periods of 8–64 h. Only when the frequency of the light cycle fell within a fixed multiple of 24 h (i.e. 24, 48, and 72 h) did plants exhibit an appropriate (SD) photoperiodic response. These ‘resonance’ protocols were later used in elegant studies of birds (white-crowned sparrow, house finch and quail), in which a 6-h light period followed by dark periods comprising multiples of 24 h (i.e. 6 h light:18 h darkness, 6 h light:42 h darkness and 6 h light:66 h darkness) resulted in a SD response (i.e. reproductive suppression). Cycles which were not delivered in multiples of 24 h (i.e. 6 h light:30 h darkness and 6 h light:54 h darkness)-induced gonadal maturation (LD response) (Hamner 1963, Follett & Sharp 1969, Follett *et al*. 1974). In this study, light is posited to fall at a photosensitive phase on alternate days, triggering a ‘LD’ response. Later studies on seasonal Syrian hamsters yielded similar results (Elliott *et al*. 1972). This now provides a framework for investigating the endocrine and molecular mechanisms underlying seasonal timing, and the challenge is to understand how changes in a circadian signalling system can be used to drive an annual cycle.

## Light input mechanisms and the rhythmic melatonin signal

Mammals have diverged from birds and other vertebrates in the way they relay light–dark information and the hormonal signals involved. In mammals, the nocturnal production of the indoleamine hormone melatonin by the pineal gland provides a crucial step in the photoperiodic relay, and removal of the pineal prevents mammalian photoperiodic responsiveness (Hoffman & Reiter 1965, Bittman *et al*. 1983, Carter & Goldman 1983, Goldman 2001). In non-mammalian vertebrates, melatonin does not play a significant role in seasonal photoperiodic responses. The eye in mammals is the only photoreceptive organ and its removal also abolishes photoperiodic responses (Reiter 1980, Nelson & Zucker 1981, Meijer *et al*. 1999). Birds and other non-mammalian vertebrates have extra-retinal and deep brain photoreceptors, therefore loss of the eyes has little effect on seasonal photoperiodic responses (Yoshimura 2013). Within the mammalian retina, rods and cones comprise the major image-forming photoreceptors, but in addition to this, there is a non-image-forming photoreceptor (melanopsin; *OPN4*), which is expressed in the ganglion cell layer. Photic inhibition of pineal melatonin persists in the absence of rods and cones (Lucas & Foster 1999), indicating a role for the non-image-forming photoreceptor *OPN4* in melatonin inhibition by light and circadian rhythms. The *OPN4* photoreceptor is involved in both circadian re-setting mechanisms and pupillary light responses (Berson *et al*. 2002, Hattar *et al*. 2002, Lucas *et al*. 2003). Knockout of *Opn4*, in mice results in loss of light-dependent suppression of arylalkylamine *N*-acetyltransferase mRNA (rate-limiting enzyme in melatonin synthesis) but only in mice that lack rod photoreceptors (Panda *et al*. 2003).

Changes in duration of the light–darkness cycle are decoded in mammals within the supra-chiasmatic nucleus (SCN) of the hypothalamus. It has been proposed that altered phasing of clock genes, *PER* (*PER1*) and *CRY*, in the SCN leads to an encoding mechanism for tracking seasonal changes in photoperiod (Nuesslein-Hildesheim *et al*. 2000, Sumová *et al*. 2003, Hazlerigg *et al*. 2005, Inagaki *et al*. 2007, Naito *et al*. 2008). This SCN read-out of the photoperiod signal has all of the characteristics of an internal co-incidence timer, and is also reflected in changes in the patterns of SCN electrical activity (Brown & Piggins 2009). The current dogma is that the mammalian pineal is controlled via a polysynaptic pathway from the SCN, relaying the light-entrained SCN rhythm into a rhythmic signal of circulating melatonin. This rhythm is characterised by a large nocturnal increase in circulating melatonin that closely reflects the duration of night period. This model is perhaps rodent-centric, as in other species there may be a role for direct photic regulation, independent of circadian input. For instance, in arctic reindeer, housed with artificial light cycles, there is an hourglass-like response to light–darkness cycles, with acute melatonin responses to dark exposure, and no evidence for an underlying circadian input (Stokkan *et al*. 2007, Lu *et al*. 2010). In such high-latitude species, the melatonin rhythm provides an accurate read-out of the prevailing photoperiod, but the endogenous melatonin cycle is only generated at times of year with a distinctive light–darkness cycle for a few months in the spring and autumn, and the rhythm dampens to extinction in the continuous darkness of the arctic winter (Stokkan & Reiter 1994).

The primary mechanisms driving seasonal reproductive change resides in the neural control of the pattern of secretion of gonadotrophin-releasing hormone (GNRH) from the hypothalamus. Pulsatile release of GNRH drives luteinising hormone (LH) and follicle-stimulating hormone from the pituitary gland, which activates the gonads. Kisspeptin is a potent GNRH secretagogue, driving most aspects of reproduction in mammals (Oakley *et al*. 2009). RFRP3 (RFamide peptide) inhibits GNRH in the sheep and hamster; however, it can also activate GNRH in the hamster depending on photoperiod (Dardente *et al*. 2014). The *GnIH* gene in birds is equivalent to RFRP and is well characterised as having inhibitory effects on the gonadotrophic axis (Tsutsui *et al*. 2013). In sheep, the transition from the breeding to non-breeding season is associated with a dramatic reduction in the frequency of GNRH pulses, suppressing the gonadotrophin drive to the gonads, resulting in gonadal regression (Lincoln & Short 1980, Goodman *et al*. 1982, Robinson *et al*. 1985, Barrell *et al*. 1992). The mass of the gonads, in mammals will change by approximately 10- to 15-fold, while in birds this is over 100-fold, in response to season (Dawson *et al*. 2001). These seasonal changes in GNRH have long been recognised in mammals as being regulated by the melatonin signal. For instance, a series of studies using pinealectomised and ovariectomised female sheep with constant-release oestradiol implants to mimic the mid-luteal phase of LH secretion reveal that artificial patterns of melatonin-mimicking summer or winter profiles were sufficient to drive the seasonal feedback effects of oestradiol on LH secretion (Goodman *et al*. 1981, 1982, Bittman *et al*. 1983, Robinson *et al*. 1985, Karsch & Moenter 1990, Barrell *et al*. 1992). Infusion of long-duration (winter-like) melatonin signals to pinealectomised Siberian hamster males blocks gonadal growth, and this effect is dependent on presentation of a regular repeated unbroken series of signals over many days (Carter & Goldman 1983, Goldman *et al*. 1984). The expression of kisspeptin and RFRP is altered by photoperiod and melatonin in mammals (Simonneaux *et al*. 2012), linking them to seasonal reproductive changes.

The role of the SCN and the circadian clock in the interpretation of the melatonin signal is less clear. Early studies of Siberian hamsters revealed that SD-like effects of melatonin are blocked by SCN lesions (Bartness *et al*. 1991), but in Syrian hamsters gonadal responses to programmed infusions of melatonin are insensitive to the lesions of the SCN, or to the phase of the light–darkness cycle at which the signal is presented (Maywood *et al*. 1990). The photoperiodic response to the melatonin signal, however, is sensitive to signal frequency, albeit in a range far greater than that which operates within the circadian system (Maywood *et al*. 1992, Grosse *et al*. 1993, Stirland *et al*. 1996).

The potential involvement of the circadian timing system has been explored further in studies of the *Tau* mutation in the Syrian hamster. Here, circadian activity cycles are accelerated from 24 to 20 h, as a consequence of a gain-of-function mutation in a key kinase (casein-kinase 1ε) involved in the phosphorylation of PER proteins (Lowrey *et al*. 2000, Meng *et al*. 2008). As a result of this mutation, melatonin rhythms in *Tau* hamsters are also generated every 20 h (Lucas *et al*. 1999). *Tau* hamsters do remain photoperiodic, but require photoperiod cycles presented in 20 h patterns in order to mount appropriate neuroendocrine responses. *Tau* hamsters cannot entrain properly to 24 h cycles, nor can they respond to short photoperiods in a 24 h cycle; in these conditions, they remain reproductively active in a ‘LD’ state. However, when maintained in continuous darkness, allowing the free-running melatonin rhythm to operate, *Tau* hamsters undergo testicular regression at a 20% more rapid rate than their WT counterparts under the same conditions (Loudon *et al*. 1998). Are such differences between *Tau* and WTs a consequence of an accelerated melatonin cycle, or has this circadian mutation also perturbed the response to the hormone? Pinealectomised *Tau* and WT hamsters were exposed to artificial infusions of long-duration (reproductive inhibitory) melatonin signals presented at a range of frequencies from 16 to 28 h. Remarkably, *Tau* hamsters responded to signals every 16 or 20 h, but were refractory to longer-frequency signals. The WT animals in contrast only responded to 24 or 28 h frequencies. This suggests that there is approximately a 4-h shift in the frequency–response function to melatonin signals, correlating with the altered circadian period. Thus, there may indeed be a genetic basis, involving the core circadian clockwork, in the interpretation of sequential melatonin signals at the target tissue (Stirland *et al*. 1996), which we consider below when we look at the circadian read-out mechanisms that operate in a melatonin-target tissue.

## Melatonin receptors: unexpected distribution and expression in the pituitary

Two subtypes of high-affinity G-protein-coupled melatonin receptors have been identified, termed MT1 and MT2. MT1 is thought to be principally concerned with photoperiodic signal transduction (Reppert 1997). MT2 has a restricted expression, being largely absent in the hypothalamus or pituitary of adult mammals, while in photoperiodic Siberian hamsters MT2 appears to be a pseudogene (Weaver *et al*. 1996). A related receptor has been identified in mammals as an orphan G-protein-coupled receptor, Gpr50 melatonin-related receptor (Reppert *et al*. 1996, Weaver *et al*. 1996). GPR50 is now identified as the mammalian orthologue of the high-affinity avian MEL1C receptor, but has undergone rapid evolution in the mammalian lineage (Dufourny *et al*. 2008) and lost its capacity to bind to melatonin. In mammals, *GPR50* expression in the brain is concentrated in circum-ventricular hypothalamic areas, adjacent to or overlapping with photoperiodic deiodinase enzyme regulation, and here, it is under strong photoperiodic regulation (Barrett *et al*. 2006). Knockout of this gene in mice leads to aberrant leptin responses and abnormal thermogenic responses to food restriction (Bechtold *et al*. 2012). The physiological function of *GPR50* in seasonal mammals remains a fascinating avenue for further enquiry.

Given the importance of melatonin in the regulation of seasonal neuroendocrine function, one logical proposition for the site of action would be the hypothalamus. In pinealectomised Siberian hamsters, lesions of the melatonin-receptor expressing dorso-medial nucleus (DMN) block metabolic and reproductive responses to long-duration melatonin infusions (Ebling & Barrett 2008, Leitner & Bartness 2011). However, comparative *in vitro* autoradiography using a radio-labelled analogue of melatonin, 2-iodo melatonin and *in situ* hybridisation studies for MT1 have mapped the sites of action of melatonin in the brain across a wide range of seasonal mammals, and remarkably have failed to localise a single common hypothalamic region (Morgan *et al*. 1994). Indeed, in some species such as ferrets and seasonal wallabies, melatonin receptors cannot be detected in the brain (Paterson *et al*. 1992, Weaver *et al*. 1996, Hinds & Loudon 1997).

**Figure 2:**
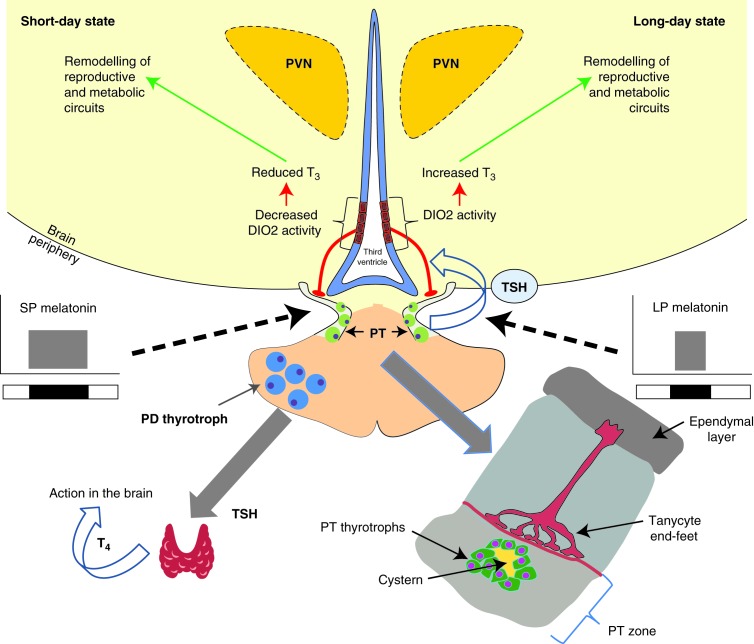
Retrograde action of TSH on ependymal cells in the hypothalamus. Photoperiod is encoded by the nocturnal melatonin signal that is sculpted by day length, generating short-duration signals in response to (long) summer day lengths. The prime site of action is the pituitary pars tuberalis. LD activation of TSHβ leads to an increase in deiodinase 2 activity in adjacent ependymal cells (tanycytes), which express the TSH receptor. This in turn leads to LD augmentation of T_3_, via conversion from T_4_. The T_3_ switch now acts on other hypothalamic circuits, leading to remodelling of reproductive and metabolic processes. Importantly, the TSH-expressing cells of the PT lack the TRH receptor, therefore cannot be regulated by a conventional hypothalamus peptide (TRH). The PT thyrotrophs are organised with an internal cistern-like structure, perhaps allowing the action of TSH on hypothalamic cells.

Unexpectedly, the pars tuberalis (PT) of the pituitary gland is a site in which melatonin binding is consistently observed in a wide range of seasonally breeding mammalian species (Morgan *et al*. 1994). The PT sits at the interface between the median eminence and the main *pars distalis* (PD) regions of the anterior pituitary (Fig. 2). Developmentally, it emerges from the rostral tip region of Rathke's pouch, and contains a mixture of endocrine cells and folliculo-stellate (FS) cells which share a number of immunological markers with brain glial cells, including GFAP and S100 protein (Hazlerigg *et al*. 2001). The PT has a distinct developmental origin from the rest of the pituitary gland, involving the bHLH transcription factor hairy enhancer of split (*HES1*) as a PT-specific differentiating factor (Akimoto *et al*. 2010). Anatomically, the long portal vessels linking the capillary bed of the median eminence to the PT run through the parenchyma of the PT. Tanycyte processes originate from the hypothalamus project to the PT (Rodríguez *et al*. 2005), while PT FS cells form cistern-like structures that make close contact with the PT–thyrotrophs, portal capillaries and tanycytes (Fig. 2) (Wittkowski *et al*. 1999). The primary endocrine cell type of the PT is thyrotrophic, expressing both the α and β sub-units of thyroid-stimulating hormone (TSH). These cells lack receptors for the hypothalamic thyrotropin-releasing hormone (TRH) (Bockmann *et al*. 1997), and do not respond to conventional hypothalamic outputs. In mammals, the MT1 receptor co-localises to the PT thyrotroph (Klosen *et al*. 2002, Von Gall *et al*. 2002, Johnston *et al*. 2006).

## Melatonin and the control of adenyl cyclase activity in the PT

The primary role of the melatonin signal is to convey information relating to the length of the day. The discovery of melatonin receptors in the PT offers a useful target tissue for further studies. Melatonin receptors are predominantly coupled to the inhibitory G_i_-protein linked with inhibition of cAMP synthesis and involved in inhibition of the classical transduction cascades induced by cAMP (PKA, CREB phosphorylation and MAPK activity; Morgan *et al*. 1989, Hazlerigg *et al*. 1991). Therefore, a model could be that nocturnal suppression of cAMP would lead to a time-release mechanism, conveying photoperiodic time. Prolonged exposure of PT cells to melatonin, however, causes sensitisation of adenylate cyclase to stimulation when melatonin is withdrawn (Hazlerigg *et al*. 1993), leading to a rise in cAMP levels at dawn (Hazlerigg *et al*. 1993, Barrett *et al*. 2003*a*,*b*). It is this dual repressive/sensitisation mode of action of melatonin that accounts for the dawn activation of some PT-expressed genes. Melatonin does not just act as a repressor, as many PT genes are acutely activated by melatonin, as discussed below (Dupré *et al*. 2008, Fustin *et al*. 2009, West *et al*. 2013).

The *Per1* transcript is expressed in the melatonin-proficient mouse strain C3H/HeN, in both the SCN and the PT of mice, and in the latter case is activated at dawn, co-incident with the decline in melatonin secretion (Sun *et al*. 1997). Analysis of *Per1* gene expression in the PT of the melatonin-deficient mouse strain C57BL/6 reveals that expression is absent, in contrast to the situation in the SCN (Sun *et al*. 1997), furthermore, pinealectomy of Syrian hamsters and C3H/HeN mice abolishes PT *Per* expression, as does deletion of the MT1 receptor in the mice (Messager *et al*. 2001, Von Gall *et al*. 2002). This indicates a dependency on melatonin for the active regulation of *Per1*. However, PT cells exposed to melatonin for 8 or 16 h show similar cAMP levels, despite having markedly different photoperiodic responses, indicating that the adenylate-cyclase-sensitising effects of melatonin are not a sufficient mechanism to explain differential responses to melatonin-signal duration (Hazlerigg *et al*. 1993, Deneubourg *et al*. 2013). Furthermore, many of the oscillating genes within the PT lack cAMP-response elements (CRE) and have no link to cAMP.

The discovery of the PT as a major site of melatonin action now presents a paradox. How might a pituitary target site be involved in remodelling of hypothalamic neuroendocrine circuits – the hallmark of the seasonal response? The answer to this lies in the remarkable discovery of thyroid hormones (THs) as key seasonal switches and their control via a novel PT pathway.

## Role for THs in seasonal timing

TH is crucially required for the expression of seasonal rhythms in multiple vertebrate species (Hazlerigg & Loudon 2008, Yoshimura 2013). This ancient signalling molecule originated well before the divergence between the vertebrate and other deuterostome lineages. TH has even been linked to the control of breeding activity in Echinoderms and the primitive chordate Amphioxus (Lancelet) (Heyland *et al*. 2005). Early studies of birds (ducks, Benoit (1936) and starlings, Woitkewitsch (1940)) demonstrate that removal of the thyroid gland dramatically altered the seasonal response of gonads. Thyroidectomy (TX) blocks many of the seasonal responses to photoperiod in the Japanese quail, and a single injection of thyroxine (T_4_) can restore the seasonal response (Follett & Nicholls 1985). Subsequent TX studies in sheep revealed that the normal transition to anoestrous at the end of the winter is blocked and can be restored by administration of T_4_ (Nicholls *et al*. 1988, Webster *et al*. 1991*a*). In TX female sheep, the mechanisms involved in oestrogen and progesterone feedback appear normal, as does the frequency and amplitude of the GNRH and LH pulses (Webster *et al*. 1991*a*,*b*).

Studies of thyroid function have been extended to other seasonal ruminants (red deer) (Anderson & Barrell 1998) exhibiting similar blockade of transition to anoestrus following TX. Sheep and deer are autumn-breeding species, with increasing day lengths in the spring terminating breeding activity. Remarkably, the effects of systemic treatment with T_4_ are only effective in terminating the breeding season in TX sheep when administered in the spring, while late summer or autumnal treatments have little effect on the onset of breeding (Billings *et al*. 2002). Further investigations of TX ewes using local constant-release micro-implants of T_4_ administered to the brain revealed that the springtime requirement for TH is localised to sites within the basal hypothalamic region (Anderson *et al*. 2003).

There may also be a role for TH transporters; MCT8 is a specific TH transporter that is regulated by photoperiod in Siberian hamsters and tanycytes in F344 rats. However, counter-intuitively MCT8 expression is increased with short photoperiods when hypothalamic tri-iodothyronine (T_3_) levels are reduced (Hanon *et al*. 2010, Ross *et al*. 2011). In F344 rats, there is an upregulation of the thyroid transporter OATP1C1 (SLCO1C1) with long photoperiods, which is consistent with increased TH with long photoperiods. Therefore, the availability of TH to the hypothalamus seems to be an important factor in driving seasonal physiology, although it is currently unclear in which direction TH transporters, such as MCT8, transport TH (i.e. into or out of the cell).

## Regulation of TH by deiodinase enzymes

**Figure 3:**
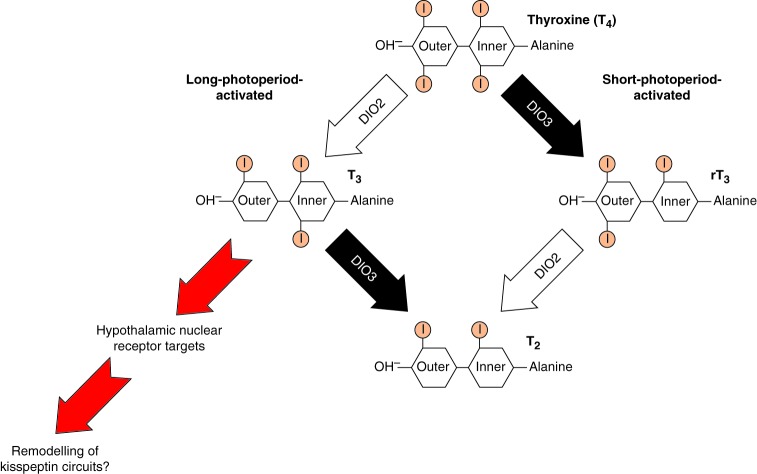
Seasonally dependent action of thyroid hormone (TH) through photoperiod-dependent changes in deiodinase enzyme expression. Thyroxine (T_4_) is the major circulating form of TH. The biological activity of T_4_ is relatively low. Upon conversion to triiodothyronine (T_3_) through outer ring deiodination, biological activity is markedly increased. This conversion to the active form is mediated by type 2 deiodinase (DIO2) in the brain. T_4_ can be converted to an inactive form, reverse T_3_ (rT_3_) by inner ring deiodination mediated by DIO3. Both T_3_ and rT_3_ can be further metabolised by DIO3 or DIO2, respectively, leading to diiodothyronine (T_2_) formation. In short photoperiod, DIO3 is upregulated leading to reduced activity of TH. In long photoperiod, DIO2 expression is increased leading to the conversion of T_4_ to T_3_, elevating bioactive TH in the hypothalamus. It is thought that altered hypothalamic T_3_ metabolism may alter kisspeptin and RFRP3 levels leading to the regulation of seasonal reproduction, although there is currently no direct evidence for this link (Simonneaux *et al*. 2013).

A key discovery by Yoshimura *et al*. (2003), working on the Japanese Quail (*Coturnix japonica*), revealed a potential mechanism to account for the seasonally dependent action of TH in the hypothalamus. Intra-hypothalamic bioavailability of T_3_, the biologically active form of TH, is governed through photoperiod-dependent changes in deiodinase gene expression (Yoshimura *et al*. 2003, Yasuo *et al*. 2005). Critically, these studies revealed that exposure to long photoperiods, which activates reproduction in quail, resulted in significant upregulation of the gene encoding the seleno-enzyme type 2 deiodinase (*Dio2*) within the ventral hypothalamic ependymal cell layer (tanycytes). These non-ciliated cells line the ventral wall of the third ventricle within the medial basal hypothalamus and have long basal processes, which terminate as end-feet in contact with the portal plexus of the median eminence (Akmayev & Fidelina 1974, Yoshimura *et al*. 2003, Rodríguez *et al*. 2005). *Dio2* removes iodine from the outer ring of T_4_, thus locally converting T_4_ to the metabolically active T_3_. In turn, exposure to short photoperiods causes suppression of *Dio2*, but upregulation of *Dio3* within these cells, which removes iodine from an inner ring of T_4_, leading to conversion to the inactive reverse T_3_ (Fig. 3). The net result is that significant local changes in concentrations of bioactive TH occur within the hypothalamus in a photoperiod-dependent manner, with elevated levels of T_3_ during long photoperiods (Fig. 3). This new model also offers insights into why responses of thyroidectomised animals to T_4_ are only effective when presented during long photoperiods, as its conversion requires LD-activated *Dio2*. T_3_ implants, in contrast, are an effective LD signal during short photoperiods (Barrett *et al*. 2007, Murphy *et al*. 2012).

The regulation of TH by photoperiod in the hypothalamus appears to be a conserved feature in several other vertebrate groups, including mammals and fishes (Hazlerigg & Loudon 2008, Nakane *et al*. 2013). There is, however, a marked species variation in the extent of LD-induced *Dio2* and SD-induced *Dio3* within the hypothalamus. In Syrian hamsters, transfer from SD to LD induces *Dio2*, with no apparent effect on *Dio3* (Revel *et al*. 2006, Barrett *et al*. 2007), while in Siberian hamsters, both enzymes are regulated, but the predominant change is SD-induction of *Dio3* (Barrett *et al*. 2007, Herwig *et al*. 2012, Prendergast *et al*. 2013). In contrast, in European hamsters and the photoperiodic rat (F344 strain), the switch from SD to LD causes increased *Dio2* expression and decreased *Dio3* (Hanon *et al*. 2010, Ross *et al*. 2011). There are also marked differences in the extent of expression of these enzymes. In short-lived rodents *Dio2* expression is limited mainly to the ependymal region (Barrett & Bolborea 2012), whereas in sheep LD-induced DIO2 is expressed within the ependymal layer, the median eminence and tuberoinfundibular sulcus (Sáenz de Miera *et al*. 2013). Importantly, these TH changes occur in the same direction in all species so far studied, irrespective of whether they are autumn-breeding (sheep and deer) or spring-breeding (seasonal rodents, quail), presenting a paradox as to how the sign of the LD-activated TH signal is reversed in species with breeding patterns timed at other times of year. Therefore, regulation of local TH bioavailability within the hypothalamus is the dominant signal-driving LD reproductive responses.

How changes in tanycyte function and altered hypothalamic T_3_ metabolism may impinge on neural pathways controlling seasonal breeding or other circuits regulating seasonal metabolic changes is less well understood, and has recently been reviewed (Bolborea & Dale 2013). As discussed earlier, there is a potential role for kisspeptin and RFRP3 in the regulation of seasonal reproduction (Simonneaux *et al*. 2013). A recent study has demonstrated that delivery of TSH in Siberian and Syrian hamsters induces DIO2 and restores Kisspeptin and RFRP expression to long photoperiod levels and reactivates the gonadal axis (Klosen *et al*. 2013). T_3_ injections administered to SD Siberian hamsters reactivated the gonadotrophic axis and led to LD levels of RF-amide peptides (Henson *et al*. 2013). This indicates that the action of TH on RF-amide neurons and subsequent seasonal control of GNRH secretion may be linked to the photoperiodic production of TSH within the PT.

## Role of the PT and TSH

**Figure 4:**
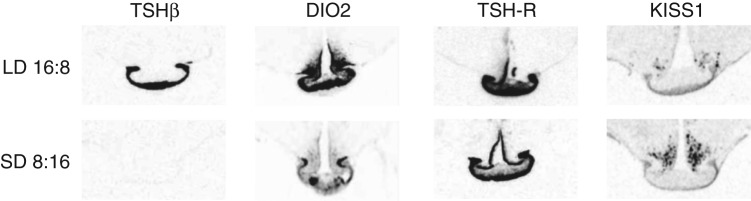
Photoperiod-controlled gene expression in the PT and hypothalamus. Autoradiographic images of radioactive *in situ* hybridisations carried out on tissue from Soay sheep with antisense probes to the β sub-unit of thyroid-stimulating hormone (*TSHβ*), TSH receptor (*TSH-R*), type 2 deiodinase (*DIO2*) and kisspeptin (*KISS1*). Sheep were acclimated under long days (16 h light: 8 h darkness) and short days (8 h light: 16 h darkness) for 6 weeks before sampling. There is a strong photoperiodic effect on pars tuberalis (PT) expression of TSHβ. *DIO2* and TSH-R expression in the median eminence (ME) and third ventricle of the hypothalamus are under photoperiodic control as is *KISS1* expression in the adjacent arcuate nucleus (ARC). Adapted from Hazlerigg & Loudon (2008), first published in *Current Biology* where images were kindly provided by E A Hanon and G C Wagner.

**Figure 5:**
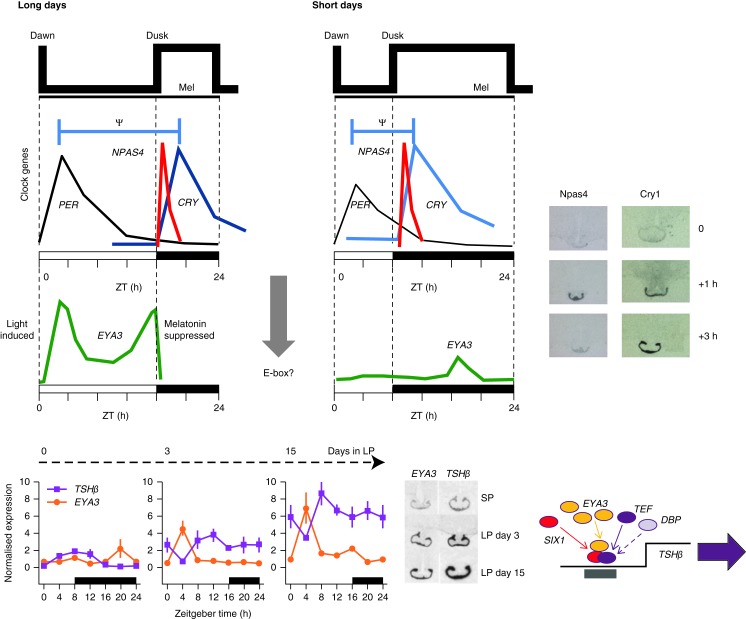
Decoding of the melatonin signal to produce a seasonal response. Decoding the melatonin signal involves changes in the temporal expression of circadian clock genes (*PER* and *CRY*). In the internal coincidence model, changes in the phase of *PER* and *CRY* gene expression are driven by the shifts in the offset and onset of melatonin secretion, such that the *PER*/*CRY* interval (ψ) varies with photoperiod. *CRY* is the probable major regulator of the PT clock, driven by rising melatonin at dusk. *NPAS4* operates as the key upstream switch, and is acutely activated by melatonin, driving the expression of *CRY*. *EYA3* has multiple E-box binding sites for clock genes, and the phase of *EYA3* expression is set by the PT clock, leading to a rise 12 h after melatonin. With short day (SD) lengths, cAMP repression by melatonin inhibits the full activation of *EYA3*. The system is de-inhibited in response to long days (LD), when *EYA3* expression is coincident with light. Thus, a clock gene rhythm and cAMP control regulate expression. *EYA3* is a strong coactivator of TSHβ expression in the pars tuberalis in synergy with *TEF*, *SIX1* and *DBP*. The lower panel illustrates the induction of *EYA3* and *TSHβ* on transfer to LD. Sheep were acclimated to 8 h light/day and transferred to 16 h light/day (LP) by acutely delaying lights off. Tissue was collected at 4 h intervals throughout 24 h on SD and the 3rd and 15th day following LD. The black horizontal bar in each graph indicates when lights were off during each sampling period. Data are mean±s.e.m. of *n*=3 animals per sampling point, with representative images from autoradiographic images of radioactive *in situ* hybridisations showing peak expression levels of *EYA3* and *TSHβ* in each of the sampling periods. The lower panel is adapted from (Dardente *et al*. 2010), first published in *Current Biology*. Data on Npas4 and Cry1 adapted from West *et al*. (2013).

Two studies in Japanese quail and Soay sheep now lend support to the concept that TSH-expressing PT cells are key regulators of hypothalamic function. In this model, PT-derived TSH acts as a local signal within the medial basal hypothalamus to regulate tanycyte *Dio2* gene expression (Hanon *et al*. 2008, Nakao *et al*. 2008). In the seasonal quail model, reproductive responses to long photoperiods are very rapid, with a rise in LH in response after exposure to a single LD. Using this protocol, Yoshimura and colleagues screened for a time course of photoperiod-activated genes following photo-stimulation, and identified TSHβ as one of two early-response genes, expressed at 14 h during the first LD exclusively within the PT. This was followed by activation of *Dio2* in the adjacent ependymal layer 4 h later (Nakao *et al*. 2008). Using i.c.v. administration of TSH, these authors demonstrated that TSH activates *Dio2*, in a cAMP-dependent manner, and initiates reproductive activation in short-photoperiod-suppressed birds. In sheep, a similar pathway operates, with LD activation of TSH and induction of DIO2 enzyme in the ependymal tanycytes of the ventral hypothalamus (Hanon *et al*. 2008; Figs 3 and 4). In both birds and mammals, the common α sub-unit is not regulated by photoperiod and remains constitutively expressed throughout the annual cycle in the PT. TSH acts on the G-protein-coupled TSH-receptor, and receptor fields for these are localised in the ependymal cell layer and also in the PT itself (Figs 3 and 4; Hanon *et al*. 2008). This also offers a new concept, whereby cAMP signalling in the PT is elevated in response to long photoperiods by a short-loop feedback of TSH on local receptors. There are striking differences in the speed of response of the TSHβ system in birds (quail) and mammals (sheep). In the former, TSHβ is activated within 14 h of the initial photo-stimulation (Nakao *et al*. 2008); this is in contrast with sheep, in which there is a sustained rise in response over 15 days (Dardente *et al*. 2010; Fig. 5). Collectively, these studies now provide a model for the seasonal regulation of deiodinase enzyme expression, involving a ‘retrograde’ action of TSH from the PT on receptor fields in the ependymal tanycytes, driving local TH metabolism in the hypothalamus.

## Role of an ancient retinal-determining gene, *EYA3*, as a LD switch

The photoperiodic induction of *TSHβ* as described earlier operates as an essential molecular switch, governing the changes in seasonal reproductive biology. But what regulates *TSHβ*? In quail, an early-response gene in PT, activated by LDs is the induction of eyes absent 3 (*Eya3*; Nakao *et al*. 2008). *Eya3* rises 14 h after the first exposure to a LD signal. In these studies, quails were exposed to an extreme photoperiod shift of from 6 h to 20 h of light, and *Eya3* expression was shown to be matched closely to that of *TSHβ*. The time course of activation of *Eya3* in birds beyond the first LD has not been defined. Other studies and our own in mammals reveal a potentially longer and dynamic time course for *Eya3* activation (Dardente *et al*. 2010, Dupré *et al*. 2010, Masumoto *et al*. 2010). In sheep, using RNA-seq, we have detected significant elevation of *EYA3* on the first full LD cycle, co-incident with the early photophase (Loudon A, Burt DW, Yu L and Wood S, unpublished observations). Similar data have been obtained in mice, with weak induction on day 1, and with a clear second peak at zeitgeber time (ZT)20 from the first day of long photoperiod. By day 3 of LD in sheep, *EYA3* is clearly induced, as is *TSHβ*, and expression levels continue to rise over the following 2 weeks, but by day 15 there is a small evening peak at ZT16 (Fig. 5; Dardente *et al*. 2010). By day 28, this second peak at the end of the photophase is of similar amplitude to the early light-phase levels (Dupré *et al*. 2010). In sheep it is apparent that *EYA3* exhibits a process of continuous dynamic activation over a period of at least 1 month following exposure to LDs, but critically, expression is confined to the photophase throughout (Fig. 5).

The eyes absent (EYA) proteins are highly conserved, from humans to insects, and were first described in relation to eye development in *Drosophila*. They are now known to be involved in the development of multiple organs (including the endocrine glands and parathyroid), innate immunity, DNA damage repair, angiogenesis, cancer metastasis and photoperiodism (reviewed in Tadjuidje & Hegde (2013)). Not only are the EYA proteins conserved but so are the regulatory networks of PAX, SIX and DACH proteins with which they interact. EYA proteins exhibit a dual role, and can act both as phosphatase enzymes and also as transcriptional co-activators (Jemc & Rebay 2007). The role of EYA3 in TSHβ regulation has been investigated using the mouse and ovine promoter sequences in NIH3T3 and COS7 cell lines respectively (Dardente *et al*. 2010, Masumoto *et al*. 2010). This revealed that synergistic activation of TSHβ is by EYA3–SIX1–TEF (Fig. 5). EYA3 lacks a DNA-binding domain and therefore acts as a transcriptional co-activator with SIX1 binding to the DNA (Xu *et al*. 1997), a mechanism of action which is not dependent on the phosphatase action of the protein (Dardente *et al*. 2010). Activation of the TSHβ promoter is, however, dependent on a D-box element with in the promoter (Dardente *et al*. 2010). Using the mouse promoter constructs, a six binding site (*So1* site) has also been identified as having an essential role in EYA3 and SIX1 activation of TSHβ. The conclusion from this study is that TEF (HLF and DBP) binds to the D-element, and EYA3–SIX1 binds to the *So1* site, activating TSHβ. Intriguingly, SIX proteins can be repressors in the absence of EYAs (Tadjuidje & Hegde 2013). In conclusion, it now appears that SIX1 is an essential co-factor for EYA3-induced expression of the TSHβ promoter (Dardente *et al*. 2010, Masumoto *et al*. 2010), directly linking EYA3 and SIX1 to seasonally regulated reproductive cycles. In this model, EYA3 is the dynamic element, with little evidence that other elements are under photoperiodic control.

A fascinating feature of this control system is that the photoperiodic induction mechanism appears to be conserved right down to the regulation of T_3_ production in laboratory mice. Detailed analysis of the D-box element reveals that the murine form is even more efficient in terms of driving TEF-dependent expression of TSHβ than the ovine D-box. Thus, it appears while mice may retain a residual photoperiodic mechanism, it fails to couple to reproductive and metabolic circuits in the brain and may perhaps be over-ridden by other (stronger) inputs such as olfactory and nutritional cues. This uncoupling, however, is likely to occur downstream of DIO2 and TH availability, as there is *Dio2* regulation in a melatonin-proficient strain of mice (Ono *et al*. 2008).

## Clock genes, the melatonin signal and the PT

The mechanisms regulating *EYA3* remain of considerable interest. Expression is tightly sculpted to the photophase on LDs, activated at dawn following the decline in melatonin signal and is acutely suppressed by melatonin at the onset of the dark phase (Dardente *et al*. 2010; Fig. 5). In the PT, the control of *EYA3* is akin to the Bünning's external co-incidence timing mechanism, which sets a ‘photosensitive phase’ and in which the circadian clock times *EYA3* expression to approximately 12 h after onset of the dark phase. Analysis of the upstream sequence of *EYA3* has identified three conserved E-boxes in the promoter, implying that it may be regulated by CLOCK and BMAL; in fact CLOCK and BMAL have an additive effect on activation of *EYA3* promoter constructs (Dardente *et al*. 2010). Although the mechanisms of dawn-activation and melatonin-mediated suppression remain to be fully identified, the pattern of induction is compatible with a role for circadian changes in cAMP activation. A current model is that *EYA3* may be regulated both by circadian E-box and CREB site activation, in a manner similar to that for the dawn-activated circadian clock gene *PER1*. Thus, in the PT, the melatonin signal sets the phase of the circadian rhythm. On SD, continued secretion of melatonin coincides with the endogenous circadian-driven rise in EYA3 12 h after melatonin onset, and EYA expression is greatly reduced due to the repression of cAMP. The system becomes de-repressed in response to long photoperiods, since the EYA3 phase is now at dawn, allowing full activation by cAMP. In this way, a circadian-regulated cycle, initiated by melatonin, drives a camp-responsive target.

The original discovery of a *Per1* transcript in the PT of mice (Sun *et al*. 1997) stimulated interest in the role that melatonin might play in the regulation of PT function. Initial studies in seasonal hamsters revealed that *Per1* and *Icer* (*Crem*) (the inducible cyclic AMP early repressor) are strongly induced in the early photophase, following the decline in melatonin (Messager *et al*. 1999). The amplitude of this response is strongly photoperiod-regulated, with lowered amplitudes in response to SD. This photoperiodic effect is also seen in Siberian hamsters, and to a lesser extent in sheep (Lincoln *et al*. 2002, Johnston *et al*. 2005). In Siberian hamsters, it carries through to changes in PER1 protein expression (Nuesslein-Hildesheim *et al*. 2000). These features have been explored more extensively in sheep, in which transcript profiling for the key transcriptional repressors PERIOD (*PER1* and *PER2*) and CRYPTOCHROME (*CRY1* and *CRY2*) were compared with the expression of the activators *CLOCK* and *BMAL1* (Lincoln *et al*. 2002). The *PER1* transcript is closely phase-locked to the early photophase. In contrast, *CRY1* is induced by melatonin (Dardente *et al*. 2003) such that its phase tracks dark onset (Lincoln *et al*. 2002, Dupré *et al*. 2008). As a consequence, the relative phasing of these two interacting components changes with photoperiod, with a relatively short PER-CRY interval with SDs, and an extended interval in response to long photoperiods (Fig. 5).

## Coincidence timing models: Bünning and Pittendrigh revisited

Changes in the coincidence of PER and CRY with photoperiod have led to the proposition that these genes might operate as an internal coincidence timer within the PT (Lincoln *et al*. 2002, 2003; Fig. 5). The close coincidence of PER and CRY during SD is consistent with this model, since these transcriptional repressors have been proposed to act as a dimeric pair, suppressing *CLOCK* and *BMAL1* expression during the circadian cycle (Reppert & Weaver 2001, 2002). This might therefore result in altered regulation of E-box-controlled genes, for which *EYA3* remains a candidate. More recently, it has become apparent that CRY proteins act as the dominant transcriptional repressor of CLOCK/BMAL1-mediated E-box transcription (Ye *et al*. 2011), and PER proteins may gate the timing of CRY nuclear accumulation. A recent study has mapped the CRY ‘cistrome’ for hepatic target genes using ChIP-seq (Koike *et al*. 2012), showing it acts on multiple targets and that only a minority of the CRYPTOCHROME-binding sites are recognised ‘clock’ elements bound to BMAL1/CLOCK heterodimers. The majority of CRY sites for instance overlap with recognition sequences for nuclear hormone receptors. CRY is therefore a prime candidate for the molecule responsible for synchronising the melatonin-driven oscillation in response to photoperiod in this endocrine tissue, but we know little of its action on target genes in the PT.

Although the internal coincidence model remains to be rigorously tested, the TSH response in *Per2* knockout mice has been measured, showing a robust photoperiodic response by *Tshβ*, *Dio2* and *Dio3* genes (Ikegami *et al*. 2013). Deletion of *Per2* altered expression of other PT-clock gene components, but importantly many of these remained rhythmic – albeit at lower amplitude. A prediction of an internal coincidence PER–CRY timer is that with very long photoperiods, the phasing of PER and CRY would become closer, and similar to the pattern observed with SD, perhaps eliciting a physiological response similar to that under SD conditions. This has been explored using sheep as a model (Wagner *et al*. 2008), and here surprisingly, transfer to ultra-long periods of illlumination (20 and 22 h of light/day) elicits responses markedly similar to those under SD conditions, with suppression of TSHβ. CRY remained locked to the onset of the short-duration melatonin signal, and PER1 after the onset of illumination. In these circumstances, with a closely associated PER/CRY rhythm, PER1 re-establishes low amplitude SD-like PER1 expression in the early photophase (Hong & Stetson 1986, Hong *et al*. 1986).

In the PT, CRY is the prime candidate for, setting the phase of the PT cycle, driven by melatonin. Typically, *CRY1* mRNA levels in the PT rise over a period 2 h or more in response to melatonin (Dupré *et al*. 2008, West *et al*. 2013). There are sites for the immediate early gene *EGR1* on the *CRY* promoter, and the EGR1-RE is acutely regulated by melatonin, but studies in cell lines indicate that *EGR1* may act as a repressor rather than an activator of *CRY* (Fustin *et al*. 2009). Using RNA-seq to define dynamic changes in the melatonin-regulated PT transcriptome, we have identified a cluster of ‘early-response’ genes, rising sharply within 1.5 h (West *et al*. 2013). Within this, a transcription factor, *NPAS4* (also known as neuronal X factor (*NXF*)), exhibits an increase of 30- to 50-fold. *NPAS4* appears to act as a key regulator of *CRY1* (Fig. 5; West *et al*. 2013). *In vitro*, *NPAS4* forms functional dimers with basic helix loop helix-PAS domain co-factors aryl hydrocarbon receptor nuclear translocator (*ARNT*), *ARNT2*, and *ARNTL* (BMAL1), transactivating both *CRY1* and also the melatonin-induced *NAMPT* promoter. The transactivation by *NPAS4*–*ARNT* appears to be co-dependent upon two conserved central midline elements within the *CRY1* promoter. *NPAS4* may therefore act as a key immediate early-response gene in the ovine PT, driving molecular responses to melatonin and setting the phase of the PT oscillation (Fig. 5).

## The PT as an integrator of seasonal hormone rhythms: the prolactin read-out

While some of the mechanisms mediating TSH regulation of TH metabolism have been mapped out, we have much less information on the seasonal control of prolactin. The hormone provides a robust read-out of a LD response in both birds and mammals (Fig. 1), and one hypothesis in mammals could involve clock-regulated changes in the classical inhibitory input from the hypothalamus to the pituitary via dopamine. Current evidence strongly indicates that this is not the case, and that instead the primary mechanism probably involves an intra-pituitary circuit and the PT. Surgical disconnection of the pituitary from the hypothalamus (hypothalamic-pituitary disconnection (HPD)) in sheep spares the pituitary and its blood supply but abrogates the neuronal input, leading to reproductive collapse due to loss of GNRH neuronal input, but remarkably the seasonal control of prolactin regulation remains robustly photoperiodic (Lincoln & Clarke 1994). These HPD sheep maintained for long periods with constant artificial LD lighting signals exhibit long-term circannual changes in prolactin secretion, but this depends on a normal LD-like melatonin signal (Lincoln *et al*. 2006). Therefore, the photoperiodic read-out is necessary for the generation of long-term oscillations – the circannual clock. This focuses attention on the PT as both an integrator of the seasonal signal, via TH regulation, and in addition as a paracrine regulator of other pituitary hormone pathways, including lactotroph function.

This concept has been further advanced by showing that the ovine PT secretes a low-molecular-weight prolactin-regulating peptide of <1 kDa – which they termed ‘tuberalin’ (Morgan *et al*. 1996). In this model, tuberalin acts via an intra-pituitary circuit to control prolactin secretion. A number of groups, including ours, have sought a candidate tuberalin (Dupré *et al*. 2010). In sheep, the tachykinin 1 (*TAC1*) gene is sharply upregulated by LD signals in the PT, and from this, neurokinin A (*NKA*) now emerges as a strong candidate for driving seasonal prolactin secretion (Dupré *et al*. 2010). *NKA* and other proposed candidates (2-arachidonoyl glycerol (Yasuo & Korf 2011)) are likely to act on intermediate cell types, as the key receptors for *NKA* (NK1, NK2 and NK3R) in the sheep PD are not observed in cells expressing *PRL* (lactotrophs) (Dupré *et al*. 2010). This indicates the involvement of indirect pathways, perhaps via FS cells, which do express neurokinin receptors.

In both hamsters and sheep, refractory responses to long-term fixed photoperiods lead to altered endocrine output, but these are known not to be driven by altered melatonin signals, which remains reflective of the prevailing photoperiod. What role might the local PT circadian clockwork play? In hamsters maintained for long periods with inhibitory SD signals, the refractory PT maintains a robust clock gene rhythm similar to that under SD conditions, reflective of the melatonin cycle, but remarkably, the production of the PT-specific prolactin releasing signal(s) reverts in such animals to a phenotype resembling that under LD conditions (Johnston *et al*. 2003). The persistence of a photoperiod-regulated PT clock gene rhythm in refractory animals has also been confirmed in sheep (Lincoln *et al*. 2005). We do not know what circadian interval-timing mechanisms are involved in prolactin regulation, but clearly *EYA3* must be considered as a candidate. A prediction would be that the expression of *EYA3* might alter in refractory states, ‘breaking’ from the prevailing photoperiod signal. Such mechanisms were never considered in the early formulations of photoperiodic models of Bünning and Pittendrigh, but one process that could be involved is epigenetic methylation-based changes, currently under investigation in our laboratories. In this regard, it is important to note that the refractory hamster model has revealed a role for epigenetic regulation of *Dio3* (Stevenson & Prendergast 2013). In sheep, there is a spontaneous decline in *DIO2* and increase in *DIO3* in animals held long term on LDs (LD-refractory), correlating with a corresponding decline in *TSHβ* expression in the neighbouring PT (Sáenz de Miera *et al*. 2013). Thus, the deiodinase signalling system is capable of spontaneous reversion in refractory animals.

We are still a long way from understanding how the circannual rhythm may be generated, and the topic has been recently reviewed (Dardente 2012), including the interesting hypothesis that one underpinning mechanism may involve seasonal histogenesis as a long-term regenerative process (Hazlerigg & Lincoln 2011). One feature is, however, however clear. In seasonal mammals, some of what we have understood from classical endocrinology needs to be revised in view of the new central role that the PT now plays. It is clearly an integrator of the circadian melatonin signal, driving hypothalamic circuits and, via a local paracrine signal, prolactin responses. In addition, the multiple pathways that control metabolic responses to photoperiod may be similarly controlled (Barrett & Bolborea 2012). The PT is also a prime candidate as a site for the generation of long-term circannual oscillations.

## Conserved pathways and evolutionarily ancient circuits

There are remarkable parallels between the mechanisms employed to time seasonal responses in birds and mammals, with both groups depending on TSH activation and a deiodinase control (Yoshimura 2013). Recent studies have yielded results indicating that the TSH system may operate as a conserved function in all vertebrates (Nakane *et al*. 2013). Fish (salmon, *Oncorhynchus masou masou*) lack an anatomically distinctive PT, but do possess a specialised circumventricular organ, the saccus vasculosus (SV), in the caudal hypothalamus of many jawed fish, which has long been known to serve as a secretory organ. A photoperiodic response in Tshβ and Dio2 protein levels in the SV of salmon was observed (Nakane *et al*. 2013), which remarkably can be recapitulated in culture by exposure to artificial lighting cycles. The SV expresses a number of opsin proteins, and removal of the SV blocks photoperiodic responses in salmon. This indicates that the TSH pathway may be well over 350 million years old, and pre-date the evolution of distinctive pituitary structures such as the PT in higher vertebrate lineages. There may indeed be links over a longer time scale. Seasonal timing is remarkably precise in many organisms, and in marine corals spawning is tightly synchronised to time of year. The *eya* gene in corals is tightly regulated by photoperiod (Brady *et al*. 2011). This raises the exciting prospect that the most ancient seasonal timers may include transcription factors, which were subsequently co-opted to drive a specific hormone-regulating pathway in vertebrates.

## Common design principles with plant seasonal interval timers

**Figure 6:**
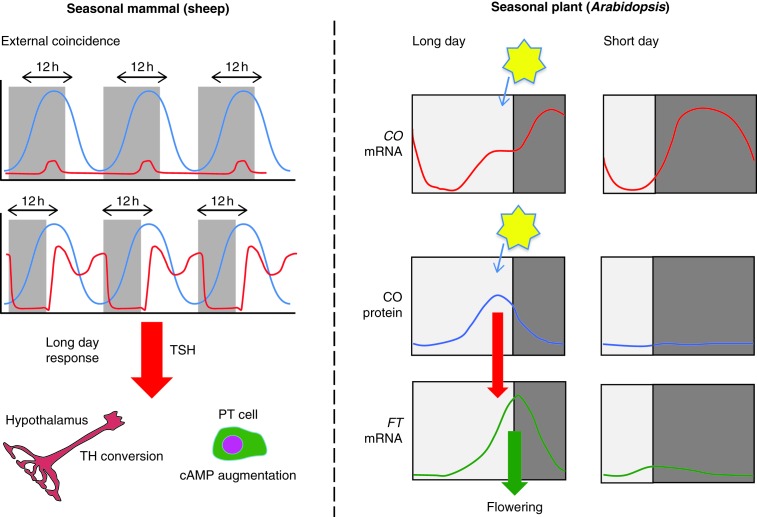
Comparison of seasonal timing by photoperiod in mammals and plants. The left panel shows a current model for an external coincidence Eya3 timer in a mammal (sheep). The blue line represents the circadian clock oscillating over a day. The grey boxes show the period of darkness, with the top graph showing a short day and the bottom a long day (LD). The red line represents *EYA3* expression. In this model, *EYA3* rises 12 h after dark onset, but is suppressed by melatonin with short photoperiods. Thus the ‘critical’ day length for activation of a LD repertoire occurs at 12 h light or more. Changes in the internal coincidence of clock genes with the onset and offset of light are proposed to drive EYA3 expression, activating expression in response to LD signalling in adjacent hypothalamic structures. Thus, seasonal timing may operate as a combination of both external and internal coincident timing processes. The right panel shows a similar coincidence timing model in a seasonal plant. Constans (*CO*) mRNA expression is under circadian control and modulated by season (red line). However, CO protein is unstable and degraded by dark exposure in plants. The protein signal can only be expressed if light is coincident with its expression. LD-activated CO then drives *FT* expression, resulting in flowering. On SD, the CO/FT system is suppressed. In both plants and animals, the key upstream activator is dark-suppressed and is released only when the phase of the internal cycle coincides with light. Data on plants were adapted from Imaizumi & Kay (2006).

The original concept of the external coincidence model of Bünning provides a crucial framework for research into the underlying genetic mechanisms driving seasonal timing. These concepts are more advanced in plants, where there are some remarkable common design principles involved (Fig. 6). The transition from vegetative to reproductive growth in plants is controlled by day length which is perceived in leaves and induces a systemic signal, called ‘florigen’, that moves through the vascular system to the shoot apex, resulting in flowering (Turck *et al*. 2008). The day length measurement mechanism in *Arabidopsis thaliana* is through the circadian regulation of the transcription factor CONSTANS (CO) by GI-FKF1, which is in turn controlled by the CRY/PER equivalents in plants (Imaizumi & Kay 2006). CO protein expression is tightly linked to the light phase and the protein actively degraded in darkness, and as a consequence, on SDs the protein is not expressed and flowering is inhibited. It is only when light is coincident with the expression of CO that the protein can be expressed – a classical Bünning model.

This looks remarkably like the EYA3–TSH system. *EYA3* transcription is acutely inhibited by melatonin and its levels only rise if light is co-incident with a phase some 12 h after dark onset. Both EYA3 and CO activate proteins that themselves act on distal targets, and it is within these target sites that we now know that other epigenetic methylation-based processes may occur, e.g. vernalisation in plants (Angel *et al*. 2011). This vernalisation process operates as a ‘salt-and-pepper’ model, gradually switching individual cells into a changed state. This type of ‘binary’ on–off signalling is now a recognised feature of stochastic control, driving pituitary gene transcription (Lionnet & Singer 2012), and is well described for prolactin (Featherstone *et al*. 2011, 2012). We believe that these mechanisms may underpin the long and sustained changes we have observed in *EYA3* activation over a period of weeks following LD stimulation.

## Relevance to human pathobiology

Despite there being little evidence for a seasonal response in the human pituitary gland, new insights into mechanisms that underlie seasonal re-programming may prove to be important for understanding pituitary pathophysiology in humans. In the human PD, many pituitary adenomas are thought to be of gonadotroph origin. Thus the mechanisms that control the programming of these cell types during seasonal switching may turn out to have significant links to the genetic programmes implicated in development or progression of pituitary adenoma. Examples of genes regulated by melatonin in the PT include epigenetic regulators such as *GADD45γ* (Schäfer 2013, West *et al*. 2013) which have been implicated in some gonadotroph adenomas in man (Zhang *et al*. 2002, Bahar *et al*. 2004, Michaelis *et al*. 2011). It remains to be established to what extent epigenetic reprogramming mechanisms overlap between seasonal responses in the PT and hyperplasia or adenoma formation in the human pituitary.

## Conclusions

The core message evolving from the recent wave of research into the genetic mechanisms driving biological timing is that the processes involved are incredibly ancient, frequently conserved, and obey a common set of rules. The more advanced modeling-based knowledge of the seasonal plant clock may provide key insights into vertebrate timing mechanisms, as we design experiments to search for the fundamental design principles involved in driving the rhythmic endocrinology of mammals. Finally, the recognition of the fascinating new role of the PT, as a retrograde regulator of hypothalamic TH action and also its paracrine control of anterior pituitary hormone secretion, now raises important questions regarding the role of the PT in the regulation of human pituitary function. This remains virtually unexplored.

## Footnote

This review is based on the 2014 Society for Endocrinology Medal Lecture, presented by Dr Andrew Loudon at the Society for Endocrinology BES 2014, Liverpool, UK.
